# Physical activity and unexpected weight change in Ontario children and youth during the COVID-19 pandemic: A cross-sectional analysis of the Ontario Parent Survey 2

**DOI:** 10.1371/journal.pone.0292934

**Published:** 2024-02-01

**Authors:** Kathryn McQuillan, Yulika Yoshida-Montezuma, Marc Jambon, Leigh M. Vanderloo, Andrea Gonzalez, Laura N. Anderson

**Affiliations:** 1 Department of Health Research Methods, Evidence, and Impact, McMaster University, Hamilton, Ontario, Canada; 2 Department of Psychology, Wilfrid Laurier University, Waterloo, Ontario, ON, Canada; 3 ParticipACTION, Toronto, Ontario, Canada; 4 School of Occupational Therapy, University of Western Ontario, London, Ontario, Canada; 5 Department of Psychiatry and Behavioural Neurosciences, McMaster University, Hamilton, Ontario, Canada; 6 Child Health Evaluative Sciences, Hospital for Sick Children Research Institute, Toronto, Ontario, Canada; Universitair Kinderziekenhuis Koningin Fabiola: Hopital Universitaire des Enfants Reine Fabiola, BELGIUM

## Abstract

The objective of this study was to investigate the association between children’s parent-reported physical activity levels and weight changes during the COVID-19 pandemic among children and youth in Ontario Canada. A cross-sectional online survey was conducted in parents of children 5–17 years living in Ontario from May to July 2021. Parents recalled their child’s physical activity and weight change during the year prior to their completion of the survey. Odds ratios (OR) and 95% confidence intervals (CI) were estimated using multinomial logistic regression for the association between physical activity and weight gain or loss, adjusted for child age and gender, parent ethnicity, current housing type, method of school delivery, and financial stability. Overall, 86.8% of children did not obtain 60 minutes of moderate-to-vigorous physical activity per day and 75.4% of parents were somewhat or very concerned about their child’s physical activity levels. For all physical activity exposures (outdoor play, light physical activity, and moderate-to-vigorous physical activity), lower physical activity was consistently associated with increased odds of weight gain or loss. For example, the adjusted OR for the association between 0–1 days of moderate-to-vigorous physical activity versus 6–7 days and child weight gain was 5.81 (95% CI 4.47, 7.56). Parent concern about their child’s physical activity was also strongly associated with child weight gain (OR 7.29; 95% CI 5.94, 8.94). No differences were observed between boys and girls. This study concludes that a high proportion of children in Ontario had low physical activity levels during the COVID-19 pandemic and that low physical activity was strongly associated with parent reports of both weight gain and loss among children.

## Introduction

Childhood obesity is a public health concern. In 2017, 30% of children 5–17 years of age in Canada were classified as overweight or had obesity, putting them at greater risk of chronic diseases including asthma, type 2 diabetes, and heart disease [1]. Low physical activity is one of many risk factors for childhood obesity [2–4]. There is considerable evidence demonstrating an inverse relationship between physical activity and body mass index (BMI) in children [5–7].

The Canadian 24-Hour Movement Guidelines for Children and Youth (5–17 years) recommend that children accumulate 60 minutes of moderate-to-vigorous physical activity and several hours of light physical activity per day [8]. In their 2022 annual Report Card on Physical Activity, a grade of a “D” was assigned to children and youth’s 24 hour movement scores as only 28% of children and youth met the guidelines, a decrease from the last two years [9].

The emergence of the COVID-19 pandemic required public health restrictions to limit the spread of the virus. Temporary cancellations of organized extracurricular activities and closures of schools, and recreation centres were widespread and disrupted daily behaviours, including physical activity and sport participation [10]. A growing body of literature indicates that public health restrictions were associated with a decrease in physical activity and an increase in sedentary time in children [11–16].

While physical activity in children and youth decreased during COVID-19 lockdowns [9, 17, 18], emerging evidence suggests that child weight has increased [12, 14, 19–21]. During the pandemic, longitudinal trends show a doubling in the rate of BMI increase compared to pre-pandemic rates [21] and significant weight gain in school-aged children [20]. Specifically, the longer the duration of school closures and reduced physical activity, the higher the predicted increase in BMI and obesity [11]. The evidence to-date suggests that COVID-19 related weight gain has been most prevalent in children already vulnerable to unhealthy weight gain [14, 19, 21]. Increases in BMI during the pandemic have been observed to be greater for boys and non-Hispanic Black and Hispanic children, than for girls, and non-Hispanic White and Asian children [11, 19]. Boys with pre-existing obesity saw the greatest increase in BMI during lockdowns [14].

Conversely, social restrictions may have caused unexpected weight loss as a result of increased prevalence of eating disorders [22]. Disruption of daily behaviours and routines may have influenced regular coping mechanisms, and depleted healthcare resources may have impacted access to eating disorder resources and treatment options [23]. Electronic health records of 5.2 million individuals, mostly in the United States, indicated that diagnostic incidence of eating disorders increased by 15.3% in 2020 compared with previous years, and occurred primarily in adolescents and people with anorexia nervosa [24].

While evidence is emerging on physical activity and weight change in children during the COVID-19 pandemic, limited literature is available within Canada. Canadians experienced restrictive public health responses to COVID-19 during the first years of the pandemic, including lengthy lockdowns and school closures [25], potentially placing children in Canada at increased risk for unhealthy weight changes and reductions in physical activity. Therefore, the objective of this study was to investigate the association between children’s parent-reported physical activity levels and weight changes during the COVID-19 pandemic among children and youth in Ontario Canada.

## Methods

### Study design and sampling

A cross-sectional study was conducted using data from the Ontario Parent Survey 2 between May 4^th^ and July 3^rd^, 2021 [26, 27]. Caregivers and parents (hereafter collectively referred to as parents) of children 5–17 years of age in Ontario, Canada were recruited to participate in the voluntary, open, online survey. Initial contact with participants was made online using various crowdsourcing methods including social media advertisements and emails sent by participating public health units, Ontario EarlyON centers, school boards, municipal, and community organizations. To increase the diversity and representativeness of the sample, a market research firm recruited an additional 730 participants. As an incentive for participating, parents had the option to enter a draw for a chance to win one of three iPads or one of fifty Amazon gift cards.

The survey was developed with feedback from community partners and pilot tested. The survey was available for completion in English or French. A secure web application, REDCap, was used for survey administration and data collection. For households with multiple children, the caregiver was asked to select the child whose birthday was closest to the survey date as the ‘target child’.

This study had research ethics approval from the Hamilton Integrated Research Ethics Board (HiREB #10583). Parents provided informed written consent. Study reporting was guided by the Checklist for Reporting Results of Internet E-surveys [28].

### Measurement of variables

The primary outcome of interest in this study was parent-reported recall of their children’s weight change during the COVID-19 pandemic. Participants were asked “Over the past year, has [TARGET CHILD]’s weight changed”, and were given the following options: “gained more weight than expected”, “lost more weight than expected”, “healthy growth or no change”, “don’t know”, or “prefer not to answer”.

There were four parent-reported physical activity exposure variables that were analyzed in this study. Moderate-to-vigorous physical activity was measured by asking “Thinking of the past week, how many days did [TARGET CHILD] exercise or participate in MODERATE to VIGOROUS PHYSICAL ACTIVITY for a total of at least 60 minutes” (such as energetic sports, running and dancing) with response options ranging from 0–7 days per week. Light physical activity was measured as “Thinking of the past week, how much LIGHT PHYSICAL ACTIVITY did [TARGET CHILD] participate in on a typical weekday” with response options ranging from <30 to >240 minutes. Outdoor play or leisure time was measured as “Thinking of the past week, how much OUTDOOR play or leisure time did [TARGET CHILD] participate in on a typical weekday” with response options ranging from <30 to >60 minutes. Lastly, parent-perceived concern about their child’s overall physical activity was measured as “Please indicate how much the statement applies to you: I am concerned about the amount of physical activity my child(ren) are getting” with response options on an ordinal scale from 1–7, where 1 = Not at all, 4 = Somewhat, and 7 = A lot.

### Statistical analysis

A complete case analysis was conducted using SAS 9.4. The frequency and proportion of respondents were provided for the outcome and exposures. Chi-squared tests were used to investigate differences between boys and girls for specific features of the study population. The adjusted odds ratios (OR) and 95% confidence intervals (CI) of the association between exposures and child weight change were estimated using multinomial logistic regression with “healthy growth or no change” as the referent group. Models were adjusted for child age and gender, parent ethnicity, current housing type, method of school delivery, and financial stability as measured by parents’ difficulty paying bills. The interaction term between each of the exposures and gender were tested separately and a p value of <0.05 to indicate statistical significance. The results were also stratified by gender; however, gender identity other than boy or girl was not shown in the regression analysis due to small numbers (<1% of the total population).

## Results

A total of 13,920 participants opened the survey and provided informed consent to participate. Of these, 9% did not respond to any questions and 7% were considered invalid and excluded from analysis (e.g., based on age or region of residence), leaving 11,778 (84%) parents who participated in the study. Of 11,778 participants, 8750 had a child between the ages of 5 and 17 years. Of 8750 parents, a total of 7270 (83%) of parents reported on their child’s weight change over the past year: 6.3% reported that their child had lost more weight than expected, 24.8% reported greater than expected weight gain, and 68.9% reported healthy growth or no weight change (Table 1).

**Table 1:** Description of study population (n = 8750).

	Missing (N)	Overall Frequency N (%)	Girls Frequency N (%)	Boys Frequency N (%)	P-value^1^
**Child Gender**	756				
Girls		4415 (51.5)	4415		
Boys		3821 (47.8)		3821	
Other^2^		58 (0.7)			
**Child Age**	840				
5–12 years		5530 (69.9)	2590 (68.8)	2900 (71.4)	
13–17 years		2380 (30.1)	1177 (31.2)	1160 (28.6)	
**Child Weight Change**	1480				0.2
Gained more than expected		1805 (24.8)	860 (24.9)	923 (24.7)	
Lost more than expected		456 (6.3)	232 (6.7)	215 (5.7)	
Healthy growth or no change		5009 (68.9)	2361 (68.4)	2605 (69.6)	
**Parent Ethnicity**	332				
North American or European		6916 (82.2)	3069 (83.0)	3259 (82.0)	
East, Southeast, and South Asian		401 (4.7)	176 (4.8)	192 (4.8)	
African or Caribbean		238 (2.8)	89 (2.4)	113 (2.9)	
All other^3^		863 (10.3)	364 (9.8)	410 (10.3)	
**Parent Education**	105				
High school or less		957 (11.1)	394 (10.4)	449 (11.0)	
Trade, College, or University		7688 (88.9)	3397 (89.6)	3627 (89.0)	
**School delivery**	734				
In-person		1604 (20.0)	769 (20.2)	827 (20.1)	
Remote (virtual/online)		1505 (18.8)	744 (19.5)	749 18.2)	
Mixed or Other		4890 (61.0)	2298 (60.2)	2529 (61.5)	
Not applicable		14 (0.17)	6 (0.1)	8 (0.2)	
**Current Dwelling**	171				
House		7867 (91.7)	3503 (92.0)	3761 (91.7)	
Low or high-rise apartment		622 (7.3)	269 (7.1)	299 (7.3)	
Mobile, hotel, group home, other		90 (1.0)	36 (0.9)	40 (1.0)	
**Difficulty paying bills**	2133				
Yes		675 (10.2)	315 (10.0)	344 (10.1)	
No		5942 (89.8)	2819 (90.0)	3063 (89.9)	
**Parent concern about physical activity**	1908				>0.01
1–2 (Not at all)		1686 (24.6)	749 (23.1)	924 (26.3)	
3–5 (Somewhat)		2594 (37.9)	1289 (39.7)	1279 (36.3)	
6–7 (A lot)		2562 (37.5)	1206 (37.2)	1316 (37.4)	
**Light activity**	1110				0.04
≤30 minutes		2281 (29.9)	1137 (31.3)	1103 (28.1)	
60–90 minutes		2133 (27.9)	999 (27.5)	1114 (28.4)	
120–150 minutes		1180 (15.4)	563 (15.5)	609 (15.5)	
180–210 minutes		734 (9.6)	339 (9.3)	390 (10.0)	
≥240 minutes		1312 (17.2)	597 (16.4)	705 (18.0)	
**Moderate to vigorous activity**	1110				>0.01
0–1 days		2417 (31.6)	1240 (34.1)	1137 (29.0)	
2–3 days		2631 (34.4)	1278 (35.2)	1329 (33.9)	
4–5 days		1587 (20.8)	712 (19.6)	864 (22.0)	
6–7 days		1005 (13.2)	404 (11.1)	593 (15.1)	
**Outdoor play**	1102				>0.01
<30 minutes		1520 (19.9)	788 (21.7)	698 (17.8)	
30–45 minutes		2005 (26.2)	966 (26.5)	1016 (25.9)	
60 minutes		1351 (17.7)	604 (16.6)	737 (18.8)	
>60 minutes		2772 (36.2)	1281 (35.2)	1474 (37.5)	

^1^P-values were calculated using chi-square tests for differences between boys and girls

^2^Other category includes transgender and “Other”

^3^All Other includes Latin, Central, South American; Middle Eastern; Oceanic; First Nations, Inuit and Metis and “Other”

Physical activity levels and parent concern about physical activity, overall and stratified by gender, are also presented in Table 1. Per national physical activity guidelines (i.e., 60 minutes of moderate-to-vigorous physical activity per day and several hours of structured and unstructured light physical activity per day [8]), only 13.2% of children engaged in at least 60 minutes of moderate-to-vigorous activity 6–7 days per week, and 57.8% of children engaged in less than 90 minutes of light activity per day. A total of 37.5% of parents reported “a lot” of concern about their child’s physical activity over the past year.

**Fig 1:**
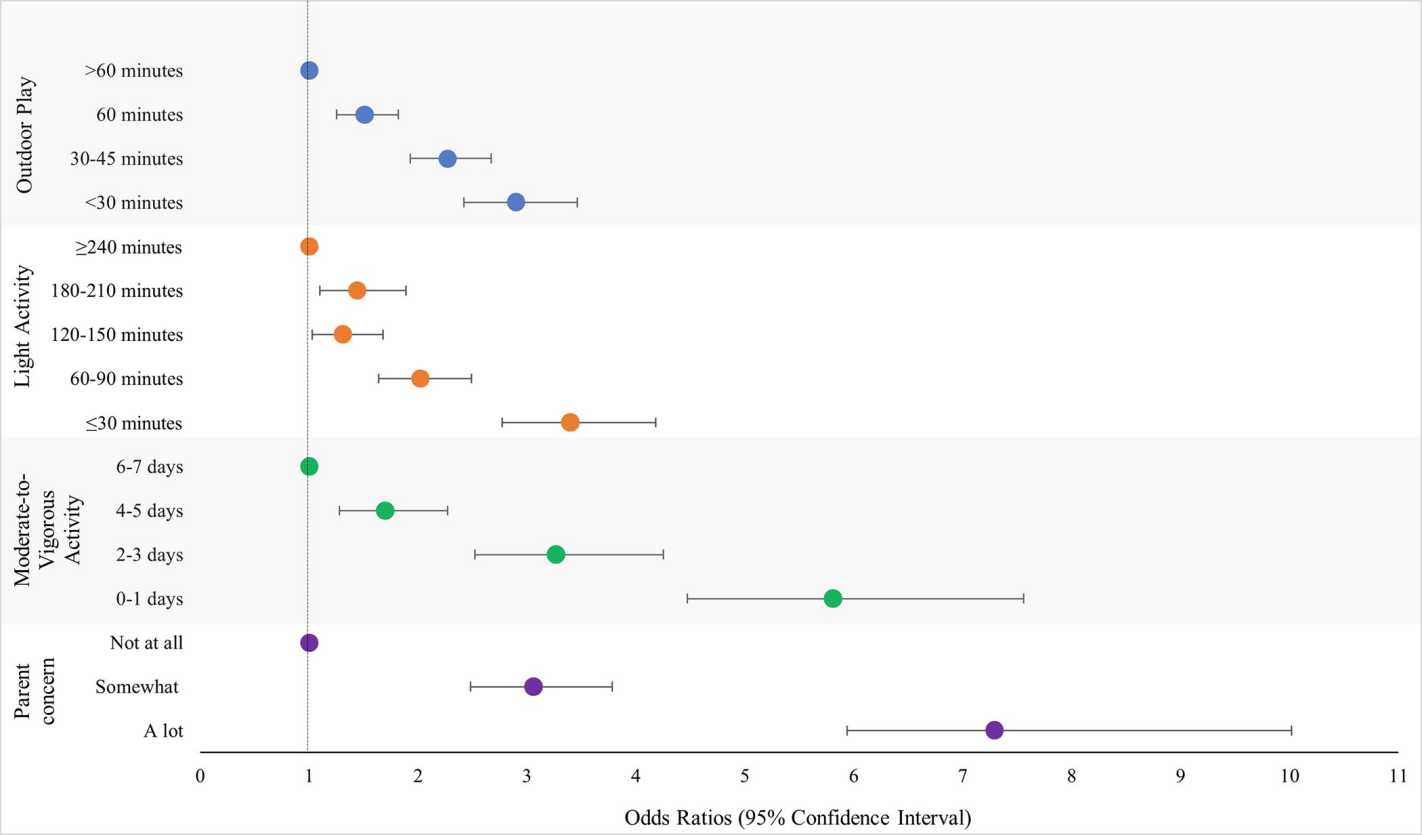
Associations between physical activity level exposures and odds of experiencing unexpected weight gain. Odds ratios were adjusted for parent ethnicity, parent education level, parent difficulty paying bills, housing type, method of school delivery, child age, child gender.

**Fig 2:**
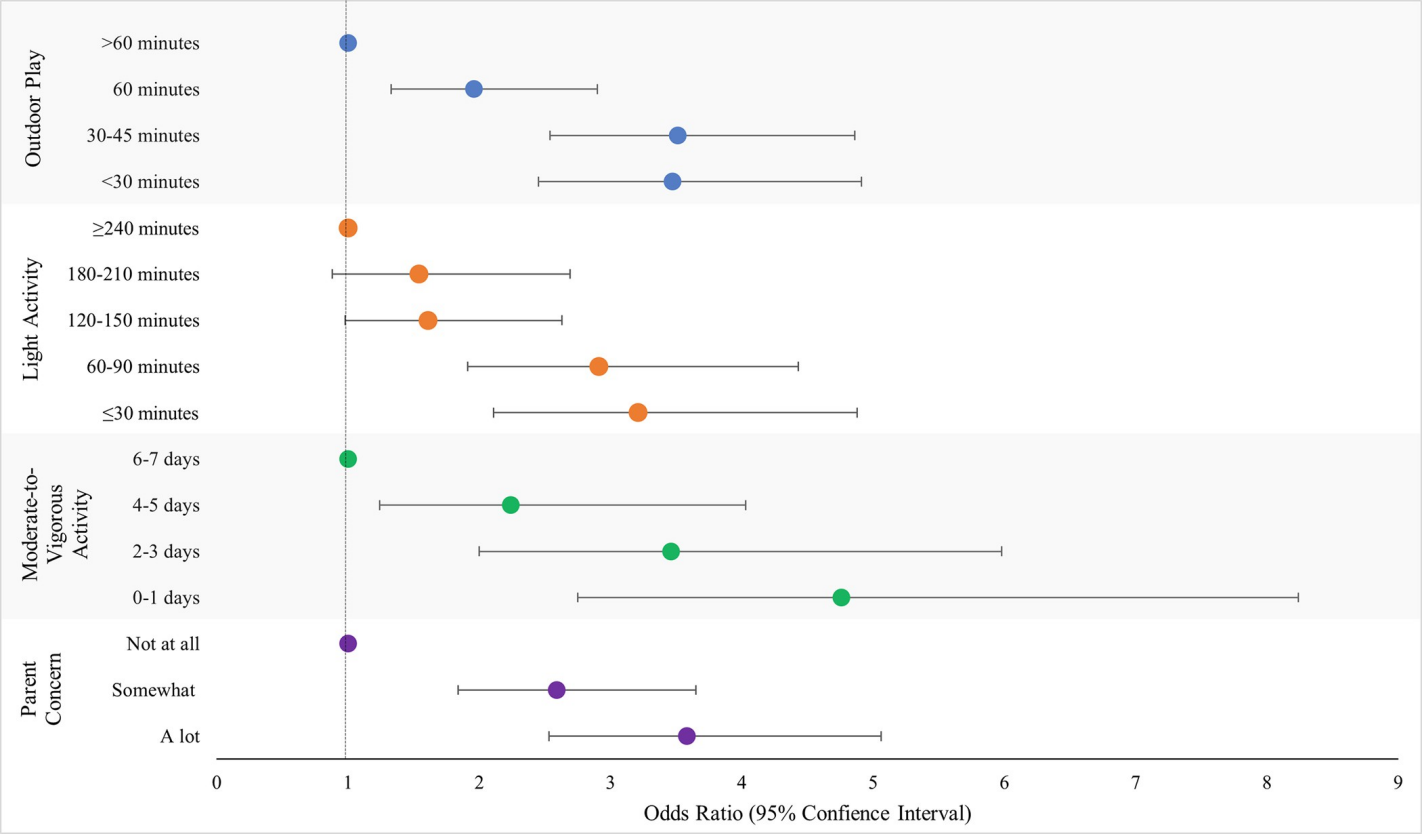
Associations between physical activity level exposures and odds of experiencing unexpected weight loss. Odds ratios were adjusted for parent ethnicity, parent education level, parent difficulty paying bills, housing type, method of school delivery, child age, child gender.

**Fig 3:**
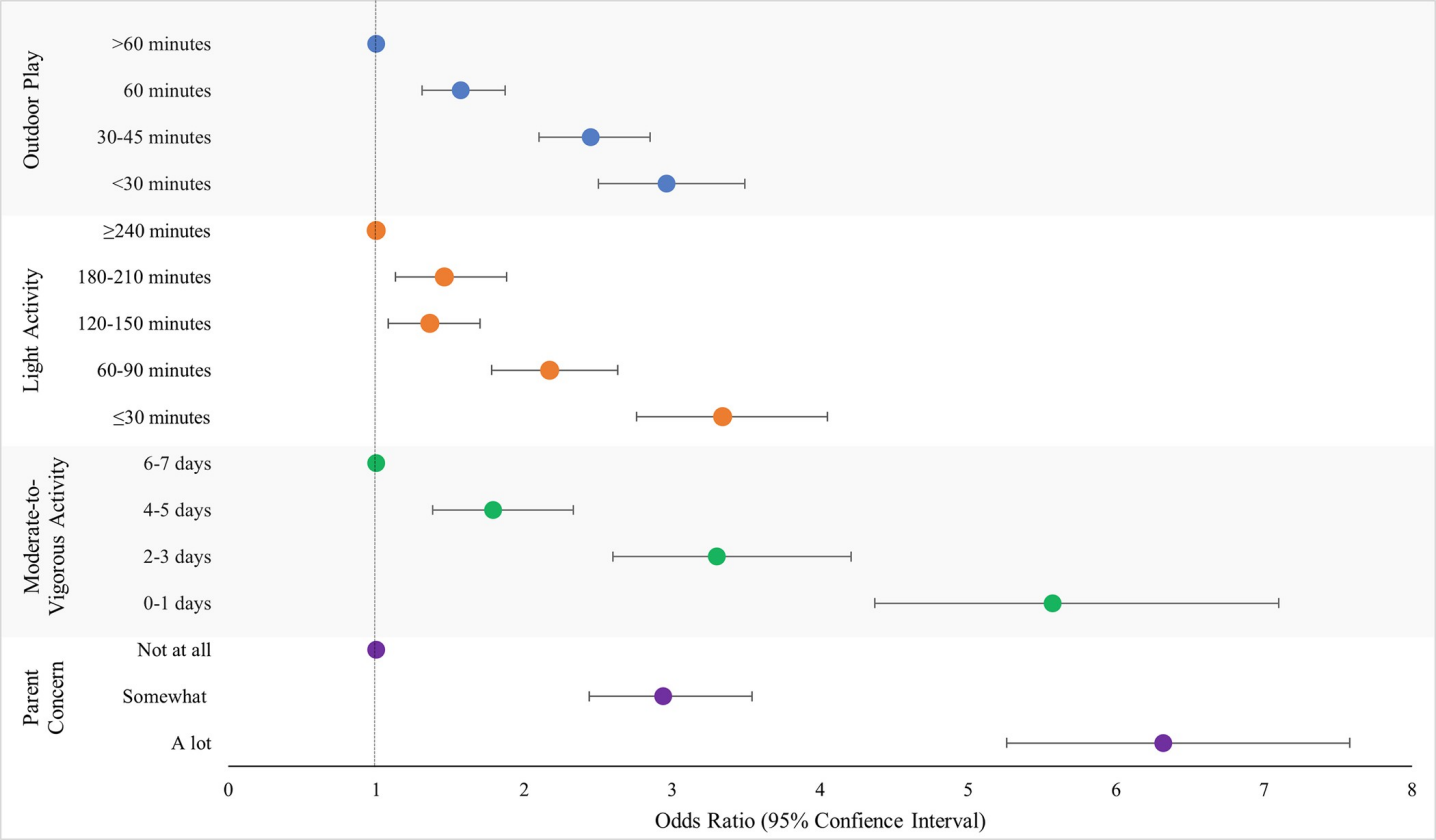
Association between physical activity level exposure and odds of experiencing unexpected weight change (weight gain or weight loss). Odds ratios were adjusted for parent ethnicity, parent education level, parent difficulty paying bills, housing type, method of school delivery, child age, child gender.

Figs 1 and 2 present the adjusted OR for the associations between physical activity related variables and child weight gain and loss, respectively. Fig 3 presents the adjusted OR for the overall association between physical activity measures and child weight change, inclusive of weight gain and loss. All physical activity variables were strongly associated with child weight gain and loss over the last year (Table 2). For each exposure a consistent ‘dose-response’ was observed such that the fewer minutes per day, days per week of activity, or greater parental concern, the greater the increase in odds of unexpected weight gain or weight loss. For example, compared to children who engaged in at least 60 minutes of moderate-to-vigorous physical activity 6–7 days per week, children who engaged in 0–1 days per week had a 5-fold increase (adjusted OR 5.81; 95% CI 4.47, 7.56) in the odds of weight gain. In all cases, the increase in odds was attenuated once adjusted for confounders.

**Table 2:** Multinomial logistic regression for the association of physical-activity related variables and child weight gain and loss compared to the reference category of “healthy growth or no change” overall and stratified by gender.

	Gained more weight than expected OR* (95% CI)			Lost moreweight than expected OR* (95% CI)			Gained or lost more weight than expected OR* (95% CI)		
	Overall	Girls	Boys	Overall	Girls	Boys	Overall	Girls	Boys
**Outdoor play**									
<30	2.90 (2.42–3.46)	2.63 (2.03–3.41)	3.25 (2.53–4.18)	3.47 (2.45–4.91)	3.59 (2.20–5.86)	3.16 (1.91–5.22)	2.96 (2.50, 3.49)	2.76 (2.16–3.51)	3.19 (2.52–4.05)
30–45	2.27 (1.93–2.67)	2.37 (1.90–2.96)	2.24 (1.76–2.84)	3.51 (2.54–4.86)	3.28 (2.04–5.27)	3.46 (2.20–5.44)	2.45 (2.10–2.85)	2.38 (1.90–2.98)	2.51 (2.03–3.09)
60 mins	1.51 (1.25–1.82)	1.48 (1.12–1.96)	1.56 (1.21–2.02)	1.96 (1.33–3.90)	2.16 (1.24–3.77)	1.76 (1.01–3.06)	1.57 (1.31–1.87)	1.57 (1.21–2.04)	1.59 (1.25–2.02)
>60 mins	1.00	1.00	1.00	1.00	1.00	1.00	1.00	1.00	1.00
**Light Activity**									
≤30 min	3.40 (2.77–4.18)	4.05 (2.96–5.55)	3.00 (2.27–3.95)	3.21 (2.11–4.88)	2.20 (1.29–3.75)	5.32 (2.59–10.89)	3.34 (2.76–4.05)	3.55 (2.67–4.73)	3.18 (2.44–4.14)
60–90 mins	2.02 (1.64–2.49)	2.11 (1.52–2.94)	2.02 (1.53–2.65)	2.91 (1.91–4.43)	2.22 (1.30–3.78)	4.15 (2.01–8.55)	2.17 (1.78–2.63)	2.14 (1.60–2.87)	2.22 (1.71–2.88)
120–150 mins	1.31 (1.03–1.68)	1.74 (1.21–2.51)	1.04 (0.74–1.45)	1.61 (0.98–2.63)	1.23 (0.64–2.34)	2.37 (1.06–5.32)	1.36 (1.08–1.70)	1.61 (1.15–2.24)	1.15 (0.84–1.58)
180–210 mins	1.44 (1.10–1.89)	1.88 (1.25–2.12)	1.16 (0.80–1.68)	1.54 (0.88–2.69)	0.85 (0.38–1.94)	3.01 (1.29–7.01)	1.46 (1.13–1.88)	1.62 (1.11–2.36)	1.33 (0.94–1.89)
≥240 mins	1.00	1.00	1.00	1.00	1.00	1.00	1.00	1.00	1.00
**Moderate Vigorous Activity**									
0–1 days	5.81 (4.47–7.56)	7.67 (4.83–12.17)	5.00 (3.61–6.93)	4.76 (2.75–8.24)	4.43 (1.99–9.87)	4.75 (2.23–10.14)	5.57 (4.37–7.10)	6.84 (4.53–10.32)	4.89 (3.60–6.65)
2–3 days	3.27 (2.52–4.25)	4.33 (2.73–6.87)	2.83 (2.05–3.91)	3.47 (2.00–5.98)	2.87 (1.28–6.44)	3.89 (1.83–8.25)	3.30 (2.60–4.21)	3.99 (2.65–6.01)	2.99 (2.20–4.04)
4–5 day	1.70 (1.28–2.27)	2.13 (1.29–3.51)	1.52 (1.07–2.17)	2.24 (1.24–4.03)	2.49 (1.06–5.82)	1.94 (0.85–4.40)	1.79 (1.38–2.33)	2.23 (1.43–3.47)	1.57 (1.13–2.19)
6–7 days	1.00	1.00	1.00	1.00	1.00	1.00	1.00	1.00	1.00
**Parent Concern**									
A lot	7.29 (5.94–8.94)	10.02 (7.14–14.06)	5.97 (4.60–7.76)	3.58 (2.53–5.06)	3.26 (2.04–5.21)	3.90 (2.32–6.55)	6.32 (5.26–7.58)	7.55 (5.69–10.03)	5.56 (4.36–7.08)
Somewhat	3.06 (2.48–3.78)	4.25 (3.01–6.00)	2.54 (1.93–3.33)	2.59 (1.84–3.65)	2.31 (1.45–3.68)	2.89 (1.73–4.84)	2.94 (2.44–3.54)	3.53 (2.65–4.70)	2.62 (2.04–3.36)
Not at all	1.00	1.00	1.00	1.00	1.00	1.00	1.00	1.00	1.00

*a were adjusted for parent ethnicity, parent education level, parent difficulty paying bills, housing type, method of school delivery, child age, child gender; OR: Odds ratio; 95% CI: 95% Confidence interval

There was no evidence of a difference in the proportion of unexpected weight gain, loss, or any measure of physical activity between boys and girls (Table 1). Overall, the dose response of increasing odds of unexpected weight gain and loss with decreasing physical activity levels was observed in both girls and boys but the stratified results did not provide strong evidence that the associations differed by gender (Table 2). Further, none of the interaction terms were statistically significant.

## Discussion

This study found that over the first year and a half of the COVID-19 pandemic in Ontario, Canada, a high proportion of children did not meet physical activity recommendations and have experienced unexpected weight changes. In this study, 86.8% of children did not meet the physical activity recommendations from the 24-Hour Movement Guidelines of 60 minutes of moderate-to-vigorous activity per day [29]. Our results are consistent with the 2022 ParticipACTION Report Card which reported that 76.2% of children (5–11 years) and 86.8% of youth did not meet the physical activity recommendation at the start of the pandemic in April 2020 compared to 82.5% of children and 88.4% of youth in October 2020 [9]. We also report that 75% of parents were somewhat or very concerned about the physical activity levels of their child. Being very or somewhat concerned about their child’s physical activity levels was strongly associated with unexpected weight gain and loss in children. Previous literature echo these findings, whereby concerned parents typically reported that children were significantly less physically active and the home environment was less supportive of physical activity compared with parents who were unconcerned [30].

The strong associations reported between all measures of physical activity and child weight gain were consistent with findings from a scoping review of pediatric obesity risk factors where irregular vigorous physical activity, low activity during break times at school, limited outdoor playtime and irregular or lack of participation in extracurricular activities were identified as risk factors for childhood obesity [31]. The results of this investigation also showed a strong association between low levels of physical activity and unexpected weight loss. A number of studies have found similar results, where underweight peers had not met physical activity guidelines, or were not necessarily participating in more physical activity then peers with obesity or overweight [32, 33]. Similarly, Ellis et al. (2017) observed that children who were underweight spent more time sitting compared to normal weight and overweight peers [34]. The results of this investigation suggest that physical activity may promote healthy growth in children and youth and is preventative against unhealthy weight loss.

Although previous literature indicates that boys tend to be more active than girls [35], the results of this investigation found that the frequency of outdoor play, light physical activity, and moderate to vigorous activity between boys and girls were very similar. Additionally, our results did not reveal a significant difference in weight gain or loss between boys and girls, despite previous literature reporting that boys more frequently have overweight or obesity than girls [11, 19, 36]. Lastly, there was no significant evidence of a difference in the association between physical activity and weight status between boys and girls. There may not have been differences by gender because boys and girls may have equally been limited by public health restrictions. Data reported by ParticipACTION also showed a reduction in differences between the proportion of girls and boys meeting physical activity recommendations as the pandemic progressed [9].

The strengths of this study include its large and diverse sample, timely data collection, and extensive measures of physical activity. However, there were several limitations. Child parent-reported weight is subject to recall and response biases, including inaccurate recall, misunderstanding of questions and a tendency to respond desirably [37]. Further, the validity of parent-reported measures of child weight *change* during the pandemic is unknown. Previous studies on child body weight perception indicate that ~44% of parents underestimate their child’s body size, and a majority of parents of children with obesity or overweight underestimate child body size [38, 39]. The magnitude of weight change and child BMI were not captured in this study, so it cannot be determined if parents’ understanding of healthy growth is consistent with child growth standards. Similarly, parent-reported physical activity levels tend to overestimate the actual physical activity behaviours of children [40]. Nevertheless, the exposure of child’s parent-reported physical activity used in this study was taken from a validated national survey [41], and has also demonstrated a weak-to-moderate correlation with accelerometer-measured physical activity in this same group [42].

The cross-sectional study design results in unknown directionality of the associations. While previous literature suggests that physical activity impacts weight change, the reverse effect may also occur, where weight gain makes physical activity more uncomfortable [43]. A pre-pandemic control group was not included in this study; thus, we cannot conclude whether the observed association between physical activity and weight change changed over time. Lastly, the results of this study may not be generalizable. Future studies should utilize a prospective design, include a pre-pandemic control, and use objective measures of weight change and physical activity to further validate the observed associations.

## Conclusion

This study found that a large proportion of children experienced unexpected weight gain and weight loss during the COVID-19 pandemic. Further, very few children during this time met physical activity guidelines. Our findings suggest a strong association between physical activity and unexpected weight change during the COVID-19 pandemic in Ontario. Although the association between low physical activity levels and weight gain is well documented, our study suggests reduced physical activity has a similar effect in increasing the odds of weight loss. Our findings contribute to the understanding of the potential impact of the COVID-19 pandemic and associated restrictions on the health and development of children and youth in Canada. Given the high prevalence of reported weight gain and weight loss in children, it is important to monitor child growth post-pandemic and identify predictors of optimal growth in children; this study suggests that interventions focused on improving child physical activity may be one approach to positively support healthy weights in this population.

## References

[1] Public Health Agency of Canada, “Tackling obesity in Canada: Childhood obesity and excess weight rates in Canada,” canada.ca. Accessed: Feb. 16, 2022. [Online]. Available: https://www.canada.ca/en/public-health/services/publications/healthy-living/obesity-excess-weight-rates-canadian-children.html

[2] Jiménez-Pavón D., Kelly J., and Reilly J. J., “Associations between objectively measured habitual physical activity and adiposity in children and adolescents: Systematic review,” *Int*. *J*. *Pediatr*. *Obes*., vol. 5, no. 1, pp. 3–18, 2010, doi: 10.3109/17477160903067601 19562608

[3] Li N. et al., “Joint associations between weekday and weekend physical activity or sedentary time and childhood obesity,” *Int*. *J*. *Obes*., vol. 43, no. 4, Art. no. 4, Apr. 2019, doi: 10.1038/s41366-019-0329-9 30705394 PMC6445763

[4] Li X. et al., “Association between Physical Activity and Age among Children with Overweight and Obesity: Evidence from the 2016–2017 National Survey of Children’s Health,” *BioMed Res*. *Int*., vol. 2020, pp. 1–8, Sep. 2020, doi: 10.1155/2020/9259742 33029532 PMC7532403

[5] Timmons B. W. et al., “Systematic review of physical activity and health in the early years (aged 0–4 years),” *Appl*. *Physiol*. *Nutr*. *Metab*., vol. 37, no. 4, pp. 773–792, Aug. 2012, doi: 10.1139/h2012-070 22765840

[6] Jurado-Castro J. M., Gil-Campos M., Gonzalez-Gonzalez H., and Llorente-Cantarero F. J., “Evaluation of Physical Activity and Lifestyle Interventions Focused on School Children with Obesity Using Accelerometry: A Systematic Review and Meta-Analysis,” *Int*. *J*. *Environ*. *Res*. *Public*. *Health*, vol. 17, no. 17, p. 6031, Aug. 2020, doi: 10.3390/ijerph17176031 32825085 PMC7503305

[7] Soares R., Brasil I., Monteiro W., and Farinatti P., “Effects of physical activity on body mass and composition of school-age children and adolescents with overweight or obesity: Systematic review focusing on intervention characteristics,” *J*. *Bodyw*. *Mov*. *Ther*., vol. 33, pp. 154–163, Jan. 2023, doi: 10.1016/j.jbmt.2022.09.004 36775513

[8] CSEP, “Children & Youth 5–17 Years: 24-Hour Movement Guidelines.” Accessed: Feb. 22, 2022. [Online]. Available: https://csepguidelines.ca/guidelines/children-youth/

[9] ParticipACTION, “2022 ParticipACTION Report Card on Physical Activity for Children and Youth,” Toronto, Oct. 2022. Accessed: Mar. 19, 2023. [Online]. Available: https://www.participaction.com/the-science/children-and-youth-report-card/

[10] Public Health Ontario, “Negative Impacts of Community-based Public Health Measures on Children, Adolescents and Families During the COVID-19 Pandemic: Update.” Jan. 11, 2021. Accessed: Apr. 16, 2023. [Online]. Available: https://www.publichealthontario.ca/-/media/documents/ncov/he/2021/01/rapid-review-neg-impacts-children-youth-families.pdf?la=en

[11] Chaabane S., Doraiswamy S., Chaabna K., Mamtani R., and Cheema S., “The Impact of COVID-19 School Closure on Child and Adolescent Health: A Rapid Systematic Review,” *Children*, vol. 8, no. 5, p. 415, May 2021, doi: 10.3390/children8050415 34069468 PMC8159143

[12] Karatzi K., Poulia K.-A., Papakonstantinou E., and Zampelas A., “The Impact of Nutritional and Lifestyle Changes on Body Weight, Body Composition and Cardiometabolic Risk Factors in Children and Adolescents during the Pandemic of COVID-19: A Systematic Review,” *Children*, vol. 8, no. 12, Art. no. 12, Dec. 2021, doi: 10.3390/children8121130 34943326 PMC8700559

[13] Koletzko B., Holzapfel C., Schneider U., and Hauner H., “Lifestyle and Body Weight Consequences of the COVID-19 Pandemic in Children: Increasing Disparity,” *Ann*. *Nutr*. *Metab*., pp. 1–3, Jan. 2021, doi: 10.1159/000514186 33498055 PMC7900479

[14] Maltoni G., Zioutas M., Deiana G., Biserni G. B., Pession A., and Zucchini S., “Gender differences in weight gain during lockdown due to COVID-19 pandemic in adolescents with obesity,” *Nutr*. *Metab*. *Cardiovasc*. *Dis*., vol. 31, no. 7, pp. 2181–2185, Jun. 2021, doi: 10.1016/j.numecd.2021.03.018 33994065

[15] Sideli L. et al., “Effects of COVID-19 lockdown on eating disorders and obesity: A systematic review and meta-analysis,” *Eur*. *Eat*. *Disord*. *Rev*., vol. 29, no. 6, pp. 826–841, 2021, doi: 10.1002/erv.2861 34460991 PMC8652707

[16] Stockwell S. et al., “Changes in physical activity and sedentary behaviours from before to during the COVID-19 pandemic lockdown: a systematic review,” *BMJ Open Sport Exerc*. *Med*., vol. 7, no. 1, p. e000960, Jan. 2021, doi: 10.1136/bmjsem-2020-000960 34192010 PMC7852071

[17] Moore S. A. et al., “Impact of the COVID-19 virus outbreak on movement and play behaviours of Canadian children and youth: a national survey,” *Int*. *J*. *Behav*. *Nutr*. *Phys*. *Act*., vol. 17, no. 1, p. 85, Jul. 2020, doi: 10.1186/s12966-020-00987-8 32631350 PMC7336091

[18] Moore S. A. et al., “Few Canadian children and youth were meeting the 24-hour movement behaviour guidelines 6-months into the COVID-19 pandemic: Follow-up from a national study,” *Appl*. *Physiol*. *Nutr*. *Metab*., vol. 46, no. 10, pp. 1225–1240, Oct. 2021, doi: 10.1139/apnm-2021-0354 34370965

[19] Brooks C. G. et al., “Pediatric BMI changes during COVID-19 pandemic: An electronic health record-based retrospective cohort study,” *EClinicalMedicine*, vol. 38, p. 101026, Aug. 2021, doi: 10.1016/j.eclinm.2021.101026 34337366 PMC8318998

[20] Chang T.-H. et al., “Weight Gain Associated with COVID-19 Lockdown in Children and Adolescents: A Systematic Review and Meta-Analysis,” *Nutrients*, vol. 13, no. 10, p. 3668, Oct. 2021, doi: 10.3390/nu13103668 34684669 PMC8540321

[21] Lange S. J. et al., “Longitudinal Trends in Body Mass Index Before and During the COVID-19 Pandemic Among Persons Aged 2–19 Years—United States, 2018–2020,” *Morb*. *Mortal*. *Wkly*. *Rep*., vol. 70, no. 37, pp. 1278–1283, Sep. 2021, doi: 10.15585/mmwr.mm7037a3 34529635 PMC8445379

[22] Linardon J., Messer M., Rodgers R. F., and Fuller-Tyszkiewicz M., “A systematic scoping review of research on COVID-19 impacts on eating disorders: A critical appraisal of the evidence and recommendations for the field,” *Int*. *J*. *Eat*. *Disord*., vol. 55, no. 1, pp. 3–38, 2022, doi: 10.1002/eat.23640 34773665 PMC8646470

[23] Cooper M. et al., “Eating disorders during the COVID-19 pandemic and quarantine: an overview of risks and recommendations for treatment and early intervention,” *Eat*. *Disord*., vol. 30, no. 1, pp. 54–76, Jan. 2022, doi: 10.1080/10640266.2020.1790271 32644868 PMC7929530

[24] Taquet M., Geddes J. R., Luciano S., and Harrison P. J., “Incidence and outcomes of eating disorders during the COVID-19 pandemic,” *Br*. *J*. *Psychiatry*, vol. 220, no. 5, pp. 262–264, May 2022, doi: 10.1192/bjp.2021.105 35048812 PMC7612698

[25] Razak F., Shin S., Naylor C. D., and Slutsky A. S., “Canada’s response to the initial 2 years of the COVID-19 pandemic: a comparison with peer countries,” *CMAJ Can*. *Med*. *Assoc*. *J*., vol. 194, no. 25, pp. E870–E877, Jun. 2022, doi: 10.1503/cmaj.220316 35760433 PMC9332918

[26] Anderson L. N., Yoshida-Montezuma Y., Jambon M., Smith B. T., Carsley S., and Gonzalez A., “Income precarity and child and parent weight change during the COVID-19 pandemic: a cross-sectional analysis of the Ontario Parent Survey,” *BMJ Open*, vol. 12, no. 12, p. e063653, Dec. 2022, doi: 10.1136/bmjopen-2022-063653 36600386 PMC9742846

[27] Zhang X. et al., “Mental Health Help-Seeking in Parents and Trajectories of Depressive and Anxiety Symptoms: Lessons Learned From the Ontario Parent Survey During the COVID-19 Pandemic,” *Front*. *Psychol*., vol. 13, p. 884591, 2022, doi: 10.3389/fpsyg.2022.884591 35783808 PMC9243663

[28] Eysenbach G., “Improving the Quality of Web Surveys: The Checklist for Reporting Results of Internet E-Surveys (CHERRIES),” *J*. *Med*. *Internet Res*., vol. 6, no. 3, p. e34, Sep. 2004, doi: 10.2196/jmir.6.3.e34 15471760 PMC1550605

[29] ParticipACTION, “The 2020 ParticipACTION Report Card on Physical Acivity for Children and Youth,” ParticipACTION, Toronto, 2020.

[30] Jackson M., Crawford D., Campbell K., and Salmon J., “Are Parental Concerns About Children’s Inactivity Warranted, and Are They Associated With a Supportive Home Environment?,” *Res*. *Q*. *Exerc*. *Sport*, vol. 79, no. 3, pp. 274–282, Sep. 2008, doi: 10.1080/02701367.2008.10599491 18816939

[31] Chi D. L., Luu M., and Chu F., “A scoping review of epidemiologic risk factors for pediatric obesity: Implications for future childhood obesity and dental caries prevention research,” *J*. *Public Health Dent*., vol. 77, no. S1, pp. S8–S31, 2017, doi: 10.1111/jphd.12221 28600842

[32] Chung A. E., Skinner A. C., Steiner M. J., and Perrin E. M., “Physical Activity and BMI in a Nationally Representative Sample of Children and Adolescents,” *Clin*. *Pediatr*. *(Phila*.*)*, vol. 51, no. 2, pp. 122–129, Feb. 2012, doi: 10.1177/0009922811417291 22315503 PMC3368285

[33] Xu Y., Mei M., Wang H., Yan Q., and He G., “Association between Weight Status and Physical Fitness in Chinese Mainland Children and Adolescents: A Cross-Sectional Study,” *Int*. *J*. *Environ*. *Res*. *Public*. *Health*, vol. 17, no. 7, Art. no. 7, Jan. 2020, doi: 10.3390/ijerph17072468 32260379 PMC7177678

[34] Ellis Y. G., Cliff D. P., Janssen X., Jones R. A., Reilly J. J., and Okely A. D., “Sedentary time, physical activity and compliance with IOM recommendations in young children at childcare,” *Prev*. *Med*. *Rep*., vol. 7, pp. 221–226, Sep. 2017, doi: 10.1016/j.pmedr.2016.12.009 28879067 PMC5575436

[35] Colley R. C., “Physical activity of Canadian children and youth, 2007 to 2015,” *Health Rep*., vol. 28, no. 10, p. 11, 2017. 29044441

[36] Cooper A. R. et al., “Objectively measured physical activity and sedentary time in youth: the International children’s accelerometry database (ICAD),” *Int*. *J*. *Behav*. *Nutr*. *Phys*. *Act*., vol. 12, no. 1, p. 113, Sep. 2015, doi: 10.1186/s12966-015-0274-5 26377803 PMC4574095

[37] Gorber C. S., Tremblay M., Moher D., and Gorber B., “A comparison of direct vs. self-report measures for assessing height, weight and body mass index: a systematic review,” *Obes*. *Rev*. *Off*. *J*. *Int*. *Assoc*. *Study Obes*., vol. 8, no. 4, pp. 307–326, Jul. 2007, doi: 10.1111/j.1467-789X.2007.00347.x 17578381

[38] Chaimovitz R., Issenman R., Moffat T., and Persad R., “Body perception: do parents, their children, and their children’s physicians perceive body image differently?,” *J*. *Pediatr*. *Gastroenterol*. *Nutr*., vol. 47, no. 1, pp. 76–80, Jul. 2008, doi: 10.1097/MPG.0b013e31815a34 18607272

[39] De La O A. et al., “Do parents accurately perceive their child’s weight status?,” *J*. *Pediatr*. *Health Care Off*. *Publ*. *Natl*. *Assoc*. *Pediatr*. *Nurse Assoc*. *Pract*., vol. 23, no. 4, pp. 216–221, Aug. 2009, doi: 10.1016/j.pedhc.2007.12.014 19559989

[40] Corder K., Crespo N. C., van Sluijs E. M. F., Lopez N. V., and Elder J. P., “Parent awareness of young children’s physical activity,” *Prev*. *Med*., vol. 55, no. 3, pp. 201–205, Sep. 2012, doi: 10.1016/j.ypmed.2012.06.021 22766008 PMC3509192

[41] Tremblay M., Wolfson M., and Connor Gorber S., “Canadian Health Measures Survey: rationale, background and overview,” *Health Rep*., vol. 18 Suppl, pp. 7–20, 2007. 18210866

[42] Sarker H. et al., “Validation of parent-reported physical activity and sedentary time by accelerometry in young children,” *BMC Res*. *Notes*, vol. 8, no. 1, p. 735, Nov. 2015, doi: 10.1186/s13104-015-1648-0 26621253 PMC4666154

[43] Prentice-Dunn H. and Prentice-Dunn S., “Physical activity, sedentary behavior, and childhood obesity: A review of cross-sectional studies,” *Psychol*. *Health Med*., vol. 17, no. 3, pp. 255–273, May 2012, doi: 10.1080/13548506.2011.608806 21995842

